# Alterations of Cytoskeleton Networks in Cell Fate Determination and Cancer Development

**DOI:** 10.3390/biom12121862

**Published:** 2022-12-13

**Authors:** Evan Ja-Yang Wang, I-Hsuan Chen, Brian Yu-Ting Kuo, Chia-Cheng Yu, Ming-Tsung Lai, Jen-Tai Lin, Leo Yen-Ting Lin, Chih-Mei Chen, Tritium Hwang, Jim Jinn-Chyuan Sheu

**Affiliations:** 1 Institute of Biomedical Sciences, National Sun Yat-sen University, Kaohsiung 804201, Taiwan; 2 Department of Surgery, Kaohsiung Veterans General Hospital, Kaohsiung 813405, Taiwan; 3 Department of Pharmacy, College of Pharmacy and Health Care, Tajen University, Pingtung County 907391, Taiwan; 4 School of Medicine, National Yang-Ming Chiao Tung University, Taipei 112304, Taiwan; 5 Department of Surgery, Tri-Service General Hospital, National Defense Medical Center, Taipei 114202, Taiwan; 6 Department of Pathology, Taichung Hospital, Ministry of Health and Welfare, Taichung 403301, Taiwan; 7 Human Genetic Center, China Medical University Hospital, Taichung 404327, Taiwan; 8 Department of Biotechnology, Kaohsiung Medical University, Kaohsiung 807378, Taiwan; 9 Institute of Biopharmaceutical Sciences, National Sun Yat-sen University, Kaohsiung 804201, Taiwan; 10 Institute of Precision Medicine, National Sun Yat-sen University, Kaohsiung 804201, Taiwan

**Keywords:** cancer, cytoskeleton, mechanotransduction, tumor microenvironment, autophagy, dormancy, immune evasion, metabolism reprogramming

## Abstract

Cytoskeleton proteins have been long recognized as structural proteins that provide the necessary mechanical architecture for cell development and tissue homeostasis. With the completion of the cancer genome project, scientists were surprised to learn that huge numbers of mutated genes are annotated as cytoskeletal or associated proteins. Although most of these mutations are considered as passenger mutations during cancer development and evolution, some genes show high mutation rates that can even determine clinical outcomes. In addition, (phospho)proteomics study confirms that many cytoskeleton-associated proteins, e.g., β-catenin, PIK3CA, and MB21D2, are important signaling mediators, further suggesting their biofunctional roles in cancer development. With emerging evidence to indicate the involvement of mechanotransduction in stemness formation and cell differentiation, mutations in these key cytoskeleton components may change the physical/mechanical properties of the cells and determine the cell fate during cancer development. In particular, tumor microenvironment remodeling triggered by such alterations has been known to play important roles in autophagy, metabolism, cancer dormancy, and immune evasion. In this review paper, we will highlight the current understanding of how aberrant cytoskeleton networks affect cancer behaviors and cellular functions through mechanotransduction.

## 1. Introduction

Cancer development has been defined as a process of accumulating genetic alterations, leading to abnormalities in cell proliferation, differentiation, DNA damage repair, metabolism, and cell death. Despite several oncogenes having been identified as cancer drivers in recent decades, emerging evidence suggests that cell–cell contacts mediated by cytoskeleton networks and mechanical cues generated by the interactions with their surrounding tumor microenvironment (TME) regulate transcriptional programs and contribute to the initiation and progression of tumors [1,2,3]. Therefore, it has been proposed that long-lived cytoskeletal structures act as epigenetic determinants of cell shape, function, and fate, even generating “memory” in cells that influences their future behavior [4]. Of note, somatic mutations and expression alterations in genes for the cytoskeleton and associated proteins are quite common in many types of cancer, supporting the viewpoint that mechanical-force-mediated signaling (also known as mechanotransduction) governed by the cytoskeleton network, should be considered one of the key elements in tissue homeostasis via its regulation of the cells’ mechanical/physical properties. These findings offer new directions for defining cancer target genes and proposing novel strategies to develop cancer treatments.

## 2. The Cytoskeleton and Its Associated Proteins Involved in Mechanotransduction

During tissue development and homeostasis, cytoskeleton proteins, including actin, microtubules, intermediate filaments, and nucleoskeleton lamins, work together to maintain mechanical architectures inside the cell. The shape and position of the nucleus are tightly controlled by the balanced forces of the cytoskeleton and nucleoskeleton. The mechanical connections maintain the cell at a highly consistent dynamic coordination, adjusting the chromatin structure to properly regulate the gene expression program [5,6,7]. Through mechanical support, cells also make connections with their surrounding microenvironment that can guide cell differentiation, proliferation, migration, and stemness formation [8,9]. Alterations in cells’ physical/mechanical properties during carcinogenesis can influence the bidirectional interactions, leading to damage to the integrity and function of the surrounding microenvironment with tumor-supporting features [10,11]. Both normal and cancer cells have machineries for detecting mechanical forces, including cell–cell [12] and cell–ECM [13] interactions. Cells respond to external forces directly through the adhesion proteins and mechanically sensitive ion channels [14]. Conversely, mechanical-force-induced ECM protein depositions can also have indirect effects on cells. For example, fibronectin may fail to form proper structures due to mechanical-force-induced unfolding [15], excess collagen fibers may suppress enzymatic degradation [16], and interactions between integrin and fibronectin may be altered by increased ECM tension [17].

**Figure 1:**
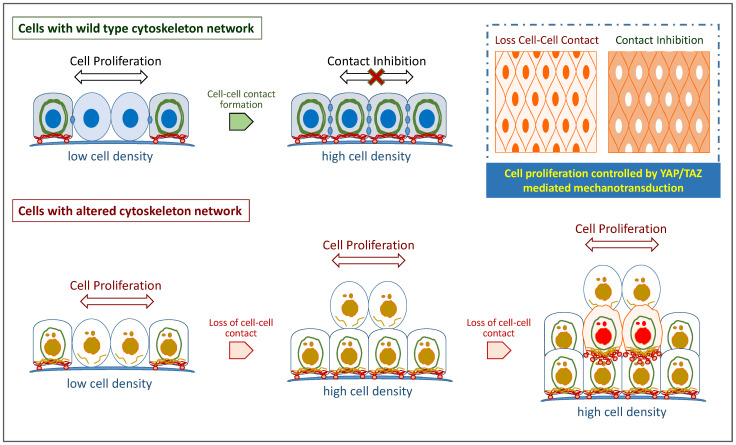
**Cell softening and cancer stemness triggered by altered cytoskeleton networks.** Cells with wild-type cytoskeleton networks maintain a mechanical structure in a symmetrical organization. When cells are at low cell density, Yap1 can be translocated into the nucleus and trigger cell proliferation. Once cells reach a high cell density with complete cell–cell contacts, the Hippo signaling is activated, and then subsequently suppresses Yap1 nuclear translocation and cell proliferation. Mutations in genes for cytoskeletal and its associated proteins disturb cytoskeleton networks, resulting in the failure of cell–cell contact formation. Thus, the cells cannot sense the contact inhibition signaling and keep proliferating continuously. Without mechanical support, cells tend to become softer and grow in multiple layers. Long-term effects of Yap1-mediated carcinogenesis will trigger cancer stemness and aggressiveness.

To transcend the mechanical signals, two major pathways were found to play important roles in these processes. The first one is chemical signaling mediated by adhesion proteins, e.g., integrins, at the cell–matrix interface [13,18]. The adhesion proteins can sense the stiffness changes in ECM, regulating the PI3K-AKT signaling pathway by activating focal adhesion kinase (FAK) [18,19]. The second pathway is the Hippo pathway, which transcends through cytoskeleton networks inside the cells [20]. When the cells sense mechanical stimuli, intermediate filament cytoskeletons and related proteins can transduce the signals from the cell membrane to the nucleus and activate downstream genes [21,22,23]. Transcription factors YAP and TAZ respond promptly to the changes in the physical tension of the cytoskeleton network, influenced by mechanical cues from the environment [20,24]. After activation, YAP/TAZ can be translocated into the nucleus, leading to upregulation of genes involved in cell cycles [12], proliferation [25], stemness formation [24,26], and tumor progression [27,28] (Figure 1). Changes in the cytoskeleton network are therefore crucial for tumor progression and metastasis, and their coordination is effectively adjusted in response to the ECM’s different characteristics, helping cancer cells to invade different tissues or penetrate through narrow pores in the ECM.

## 3. Alterations in Cells’ Physical Properties Promote the Creation of a Procancer Microenvironment

During cancer development, cells within tissues are exposed to a high degree of heterogeneity with different mechanical properties. To adapt and survive in the tissue microenvironment, cancer cells actively respond to the mechanical perturbations from extracellular matrixes, blood vessels, stroma cells, and other tumor-infiltrating cells. The biological effects of mechanical forces on cancer cells were first noted by Helmlinger et al. in 1997, when they found that mechanical stress accumulated in tumor tissue may influence tumor growth [29]. Since the continuous growth of cancer cells leads to the crowding of cells in the tissue, there is inevitably competition between cell groups to obtain growing space and nutrients, which further guides cancer evolution and clonal selection. Conversely, when a tumor is subject to mechanical stress, it also promotes deposition of extracellular matrix (ECM) proteins, resulting in an increase in tissue stiffness. Several matrix digestion enzymes, e.g., metalloproteinases (MMPs) and serine-/cysteine-proteases, can be secreted by cancer cells to degrade surrounding tissues. Stromal cells are therefore recruited from the surrounding tissue, further altering the stromal composition of the tumor. The increased ECM stiffness can lead to obstruction of blood and lymph flow, and impede the delivery of oxygen, drugs, and immune cells [30,31,32,33]. When the interstitial fluid’s pressure increases, this forces the fluid to leak from the tumor sites into the surrounding tissues. Cancer cells need to optimize their growth and stemness potentials by changing gene expression profiles in response to alterations in tissue stiffness [4,34,35].

During adaption, changes in the distribution of cytoskeleton filaments and adhesion proteins are necessary to alter cellular morphology and migration ability [36]. For example, nuclear deformation has been found to potentiate cancer cell penetration through narrow tunnels, a mechanism that has been suggested as an important driving force for cancer development and metastasis. Thus, external mechanical forces can serve as a selective barrier to screen out cell clones with a highly invasive ability to invade neighboring tissues [37,38]. Significantly, ECM deposition and structural remodeling can affect interactions between cancer cells and the ECM, activating novel signaling pathways involved in the epithelial–mesenchymal transition (EMT), metabolism reprogramming, cancer metastasis, and drug resistance [11,37,38,39,40]. Studies in mechanobiology have revealed that cancer cells exhibit quantitatively different biophysical properties from their normal counterparts with less cell stiffness and more elasticity [41,42,43,44]. Another study has also indicated that Ha-RasV12-induced transformation is associated with cell softening and loss of stiffness sensing [44]. Using 3D tumor organoids as the model, softness has been found to be a required characteristic for cancer cells to stretch beyond the tumor body and form “invasive tips” [45]. Such a mechanism is quite important for cancer cells to break away, allowing cells to move through gaps within tissues or vessels, and start to metastasize.

**Figure 2:**
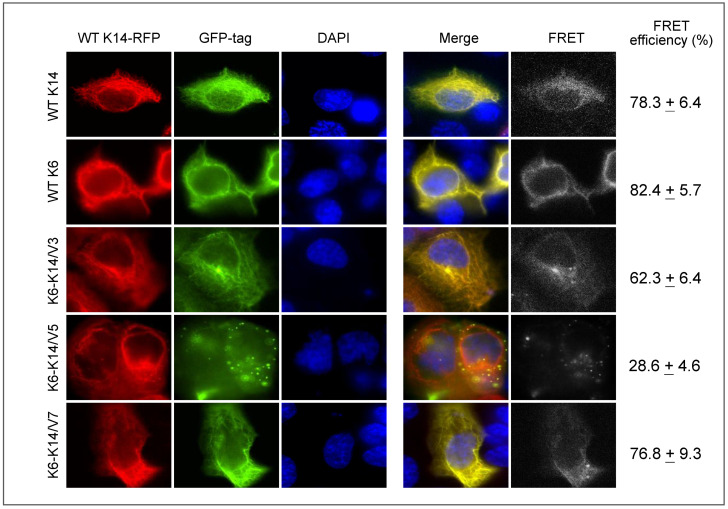
**Functional impacts of cytokeratin fusion variants on wild-type (WT) K14 networks.** K6-K14 cytokeratin fusion variants (tagged with GFP) and WT K14 (tagged with mCherry) were cotransfected into Cal-27 oral cancer cells. Permeable DAPI was utilized to stain the nucleus (blue). WT K6 and WT K14 tagged with GFP were utilized as reference controls. The representative images were taken 36 h after transfection. The interaction between each fusion variant and WT K14 was analyzed by fluorescence resonance energy transfer (FRET) assay. These data were reproduced from the original paper [51].

Studies have also shown that when normal cells undergo transformation, reorganization of cytoskeleton networks usually occurs, which affects cell adhesion and increases cell migration and invasion abilities [46,47,48]. In epithelial cells, cytokeratins can be assembled into intermediate filament (IF) networks, which provide cells with physiological strength against mechanical stress. In addition, the nuclear array formed by IF assembly is critical for cells to maintain the integrity of the nuclear envelope and control nuclear mechanotransduction [49,50]. Interestingly, a recent study reported the existence of novel cytokeratin fusions in oral squamous cell carcinoma, resulting in cytokeratins with a truncated head domain (critical for heterodimer formation and downstream signaling) or chimeras with double rod domains (critical for filament assembly and elongation) [51]. When expressed in the cells, these fusion variants can “hijack” wild-type cytokeratin networks and reduce the formation of the nuclear array, leading to nuclear deformation and aggressive cancer phenotypes (Figure 2). Similarly, specific signatures of actin filament networks were found useful for detecting the EMT statuses of the cells during carcinogenesis [52]. Somatic mutations, gene fusions, and copy number alterations (deletions and amplifications) were also detected in actin genes in several cancers with a tendency of cancer-type specificity [53]. Aberrant expression of actin subunits can confer cells with greater proliferation ability, increased migratory capability, and chemoresistance through incorporation into the normal cellular F-actin network and altered protein–protein interactions for actin binding [54]. Genetic alterations and mutations in tubulin genes were reported to be associated with drug resistance, especially against antitubulin agents [55,56,57]. Notably, a pan-cancer study revealed that copy number alterations in genes of motor proteins, including kinesin, dynein, and myosin family members, were shown in 47%, 49%, and 57% of cancer patients, respectively [58,59]. The same findings were also applied to other microtubule-associated proteins [60]. Although the mechanisms underlying these alterations in cytoskeleton genes remain poorly understood, more studies may provide insights to map the structure–function relationships.

## 4. New Cancer Genes Defined by Dysregulations in Mechanotransduction

In the past decades, cancer studies have mainly focused on signaling transduction and cell biology. To construct a unified framework for understanding different cancer behaviors, Dr. Hanahan and Dr. Weinberg were the pioneers in proposing eight key biological processes that describe the functional properties of cancer cells [61]. These include sustaining proliferative signaling, evading growth suppressors, resisting cell death, enabling replicative immortality, inducing angiogenesis, and activating invasion/metastasis. These biological features are of great help in defining cancer on the cytological front, and have allowed tremendous progress in modern cancer research and new drug development. As the cancer genomics project led by The Cancer Genome Atlas (TCGA) progresses, more and more genetic aberrations associated with cancer progression have been recently identified [62]. To effectively understand how these aberrations influence cancer development, many scientists have begun to define true drivers from many passenger alterations with several selection criteria [61,63]. Based on the oncogenic processes described above, somatic mutations in genes for the cytoskeleton and its associated proteins have been long considered as byproducts, rather than the main driving forces, during cancer development and clonal evolution.

**Figure 3:**
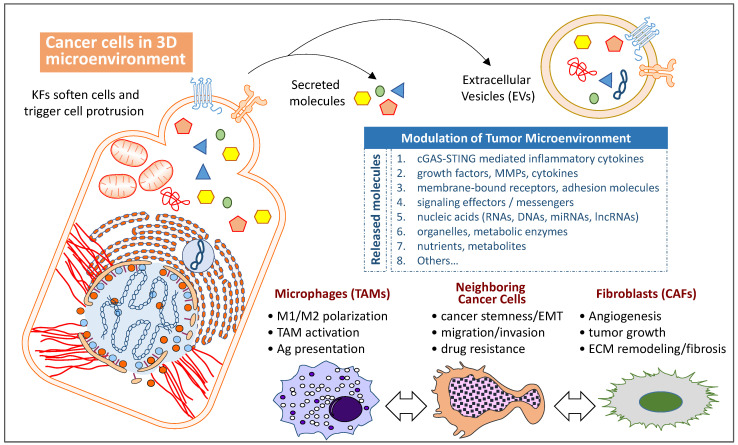
**Modulation of the tumor microenvironment (TME) is associated with alterations of cytoskeleton networks.** Cells with altered cytoskeleton network show softer mechanical properties and higher cancer stemness, which is associated with frequent membrane protrusion and extracellular vesicle (EV) release. When oxygen and nutrient gradients are present, the TME within the 3D lesions can be influenced by many factors and cells. The main remodeling factors, either released by direct secretion or embedded EVs, modulate cellular functions and behaviors of neighboring cancer cells, tumor-associated macrophages (TAMs), and cancer-associated fibroblasts (CAFs). In addition, altered cytoskeleton networks in cancer cells trigger DNA damage, leading to cGAS-STING activation and associated inflammation responses in cancer lesions.

In recent years, TME remodeling has gained more attention than before; it has been proven to be one of the key factors in regulating tumor progression [10,11]. Interestingly, both tissue stiffness surrounding the tumor and structural density of the cytoskeleton network in cancer cells have been recently suggested as another classification method for tumor staging that can score the malignant potentials of tumor lesions [64,65,66,67,68]. These findings may explain why many cytoskeletal proteins and related binding proteins are found to be mutated in cancer tissues. In particular, the cytoskeleton, nucleoskeleton, and linking complex (known as LINC complex) play a key role in connecting the nucleus to the cytoskeleton. When cytoskeletal proteins are mutated, nuclear deformation and chromosome segregation defects are caused by distortions in the LINC structure, which in turn can activate a series of mechanisms related to carcinogenesis. One well-known example is mutations in the canonical Wnt signaling pathway, e.g., β-catenin or APC, which even occur before other known driver mutations [69]. This finding highlights the active roles, but not evolutional byproducts, of changes in cellular mechanical properties during cancer onset and malignant transformation. Mutations in genes involved in cytoskeleton reorganization should be regarded as critical genetic events during cancer development (Figure 3).

## 5. Epithelial–Mesenchymal Transition (EMT) Induction by Deformations of Cytoskeleton and the Associated Proteins

When transformed cells start to switch from a nonmotile to a motile phenotype, physical alterations need to be transduced into biochemical responses that guide cellular behaviors. A well-known phenotype associated with cytoskeleton network reorganization in cancer cells is the EMT [70,71], which is also involved in the development of resistance to chemotherapy/radiotherapy [72,73]. EMT is a multifaceted program of phenotypic changes, featuring a loss of cell–cell junction proteins, e.g., desmosome or occluding [74], and a remodeling of the intermediate filament network, e.g., reduced cytokeratin filament with increased vimentin network [75,76]. Among the three different types of cytoskeleton, IF is considered to be the major contributor to providing cells with mechanical support to maintain cellular architecture and cell/nuclear shape [49,50]. As compared to actin filaments and microtubules, which are highly conserved throughout cell types, cytokeratin expression shows more diverse profiles with tissue- and cell-type specificity. More importantly, cytokeratin structures can be organized via actin filaments and microtubules, providing more mechanical support to maintain cytoskeletal geometry [77]. Previous studies have shown that the cytokeratin filament assembly process in cancer cells is distinct from its normal counterpart, and more consistent with the expression program in epithelial stem cells, and this has been suggested as another tumor staging method for cancer prognosis. These alterations include abnormal upregulation of K19 [78,79], K14 [80,81], K15 [82], K6 [83], and K17 [84,85], as well as downregulation of K8/K18 [86]. Interestingly, cytokeratin gene fusion events have recently been reported, which can promote cells undergoing the EMT process through hijacking/downregulating wild-type cytokeratin networks [51]. In particular, such gene fusion events can even be detected in normal-like epithelia adjacent to tumor counterparts, suggesting changes in mechanical/biophysical properties as an early event and a potent driving force in carcinogenesis.

Notably, many well-known oncogenic signaling molecules, such as PIK3CA and β-catenin, are also annotated as cytoskeleton-associated proteins in cells. Many somatic mutations in these binding proteins are defined as driver mutations with hotspot/recurrent mutation features [87,88,89], and thus they are substantially involved in the regulation of cytoskeleton reorganization and oncogenic signaling cascades. Based on this rationale, Gracilla et al. recently identified another cytoskeleton-binding protein named MD21D2, which also harbors a recurring activating mutation, Q311E, in several types of cancer [90]. Interestingly, the MD21D2 protein was previously defined as a member of the Mab21 domain-containing protein family, which is involved in embryonic development [91,92], DNA repair [93,94], apoptosis [95,96], and inflammatory response [97,98], the signaling pathways that are closely related to cancer development. In addition, the protein–protein network analysis placed MB21D2 at the center of the interactome formed by Mab21-containing proteins, suggesting that MB21D2 acts as a signaling hub in regulating several important cellular processes. MD21D2 overexpression or the Q311E mutant variant can activate FGFR-mediated RAS oncogenic signaling pathway, leading to cell EMT or metastasis [90].

Through EMT, individual primary tumor cells can acquire the ability to disseminate from the original site and initiate new tumors at distant organ sites [99]. Alternatively, cancer cells with partial EMT signatures can travel together as cell clusters, known as collective cell migration, to form secondary lesions [100,101,102,103]. Cell migration during metastasis is a dynamic motion; cancer cells often switch between static and migratory behaviors during individual or collective cell migration. A continuum of morphological variety provides phenotypic heterogeneity and plasticity for cancer cells to optimize their migration processes and better adapt to different ECM microenvironments [104,105]. To achieve this, expression of integrins on the surface of cancer cells can be also changed, including downregulation of integrin α6β4 (hemidesmosome) that facilitates cell adhesion to the basement membrane via laminins [106], as well as upregulation of the integrins α5β1 and α1β1 that bind to interstitial ECM proteins such as fibronectin [107] or collagen I [108]. Notably, integrin-mediated sensing, stiffening, and ECM remodeling are key steps in cancer progression, suggesting that integrins are important mechanoreceptors [18]. One of the key central proteins involved in focal adhesion formation and integrin signaling, talin-1 as a mechanosensor, has been previously reported to be overexpressed [18,109] and activated (hyperphosphorylated) [110,111], leading to increased cancer aggressiveness via converting mechanical signals into chemical signaling cascades [18,112].

## 6. Regulation of Immune TME by Mutations in Cytoskeleton-Associated Proteins

Although E-cadherin downregulation and N-cadherin upregulation are well-addressed biomarkers in EMT-induced individual cell migration [113,114], the mutational landscape in adherent junction proteins during cancer development has still been relatively unknown until a recent study on melanoma [115]. Based on a systematic investigation of classical adherent junction proteins, Korla et al. found that mutations in type II cadherins (for heterophilic interactions) are more frequent than in type I genes (for homophilic interactions) during melanoma development. Such alterations can trigger cancer growth and lymph node invasion by regulating Hippo and β-catenin/Wnt pathways. Interestingly, mutations in these junction proteins show high neoantigen potentials, which is correlated with T-lymphocyte infiltration and better clinical outcomes after immune therapy with immune checkpoint inhibitors (ICIs). Similar immune regulatory effects have also been reported in other studies, namely, that somatic mutations in atypical cadherin proteins, e.g., members of the FAT family, can promote cancer development, while patients with such mutations usually show higher mutation burdens, longer survival times, or better therapeutic outcomes by ICI therapy than patients without [116,117,118].

Inside the cell, the cytoskeleton network provides the necessary mechanical support to keep the cell nucleus intact and ensure proper chromosome segregation during cell division. Deformation changes in cells’ architectural structures or mechanical properties give rise to internal forces balancing external loads, leading to mechanical stresses. During carcinogenesis, a continuum of mechanical pressures increases, which leads to genome instability in cancer cells, resulting in loss of heterozygosity (LOH), abnormal/multipolar cell division, DNA breaks/rearrangement, micronucleus formation, and DNA copy number variations [119,120]. These genetic events create DNA fragments inside the cells that mimic viral infection. In fact, a high concentration of cytosolic dsDNAs is a hallmark for aggressive tumors, and this can subsequently activate DNA-sensing cGAS-STING signaling, leading to chronic inflammation conditions [97]. This finding suggests a molecular link that converts mechanical stress into cancer phenotypes, via long-term exposure and selection of DNA damage/sensing responses. Type I IFN is the downstream effector of the cGas-STING pathway, which subsequently triggers cell senescence, autophagy, or cell death. Secretion of related cytokines, known as senescence-associated secretory phenotype (SASP), can further activate innate immunity and T-cell priming, leading to more T-cell infiltration within tumor lesions (hot tumors) with favorable therapeutic outcomes [121,122]. Clinical studies have confirmed that chronic inflammation/long-term cGAS-STING activation caused by genome instability actually functions as a driving force to enrich cell clones with more aggressive/stemness phenotypes [123,124]. Defects in the DNA-sensing pathway are thus considered to be a mechanism by which cancer escapes immune surveillance, and downregulation of cGAS-STING signaling has been found to be correlated with poor clinical outcomes [121,125,126].

## 7. ECM Stiffening Reprograms Cancer Cell Metabolism Leading to Autophagy-Mediated Clonal Selection/Evolution

**Figure 4:**
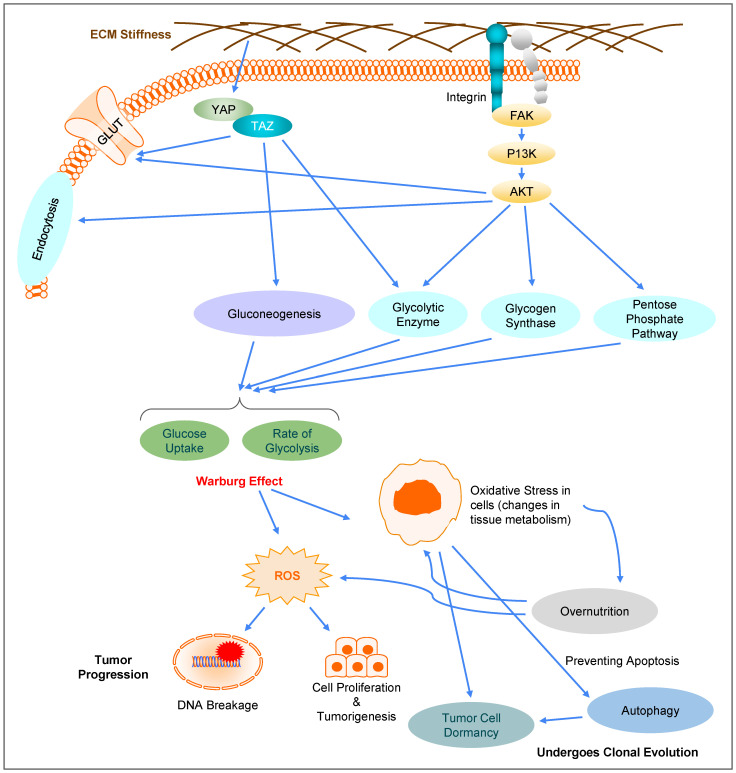
**Involvement of mechanotransduction in metabolism reprogramming and cancer cell evolution/clonal selection.** Alterations in ECM stiffness influence the glucose metabolism in cancer cells through mechanotransduction. Activation of the YAP/TAZ and integrin-FAK-P13K-AKT pathways can enhance glucose uptake and glycolysis via endocytosis, glucose transportation by GLUT, gluconeogenesis, activation of glycolytic enzymes or glycogen synthase, and upregulation of the pentose phosphate pathway. Due to the Warburg effect in cancer cells, active glycolysis can generate ROS and free radicals, leading to oxidative stress, which in turn can change cellular metabolism into a cycle of overnutrition. On the other hand, ROS can cause DNA breakage and genome instability, which serves as a driving force for uncontrolled cell proliferation, and tumorigenesis. Oxidative stress also leads to autophagy and cancer dormancy, where autophagy aids the survival of dormant cancer cells and allows cancer cells to undergo clonal evolution.

Recent studies have shown that the stiffness of ECM and nutrient metabolism in tumor cells are interdependent [127,128]. When cancer cells grow on hard substrate or migrate through tight spaces, the rate of cellular metabolism increases [129]. On the other hand, when tumor metabolism is downregulated by drugs, fibrosis can be also suppressed, resulting in reduced stiffness of surrounding tissue [127,130]. A well-established case is the involvement of ECM stiffness in changes in glucose metabolism in cancer cells. Several pathways have been shown to potentiate the process, including the YAP/TAZ and integrin-FAK-PI3K-Akt pathways, two key pathways involved in mechanotransduction (Figure 4). Others include the TXNIP, Rho/Rock-actin cytoskeleton, Rho/Rock-PTEN, GSK3, and AMPK pathways [131]. These pathways are responsible for controlling a myriad of factors, including a number of glucose transport proteins present on the cell membrane. The YAP/TAZ pathway in particular has been proven to be abnormally active in cancer cells [132]. The integrin-FAK-P13K-Akt pathway, through changes in glucose metabolism, contributes to regulating survival, apoptosis, and tumorigenic capacity of cancer cells. When activated, these pathways can increase glucose uptake and the rate of glycolysis [133], in line with the Warburg effect. This can in turn induce oxidative stress, leading to alterations in tissue metabolism, particularly in the liver, adipose tissue, pancreatic β-cells, and skeletal muscle. The interactions between the dysfunctional tissues and nutrients can then start a destructive cycle of overnutrition and oxidative stress [134,135]. In addition, oxidative stress also triggers tumor cell dormancy [136] and autophagy to prevent cell apoptosis [137], which promotes the survival of dormant cancer cells and increases the chances of metastatic tumor recurrence [138].

Cancer recurrence starts from the dissemination of cancer cells from the primary tumor site. The modern practice to treat primary tumors is surgery, followed by chemotherapy or immunotherapy. While this operation usually clears the body of the cancer cells at the initial site, cancer cells have often already metastasized to a different part of the body and gone into a quiescence state known as cancer dormancy. Emerging evidence suggests the existence of an interplay between intracellular as well as extracellular biochemical and mechanical cues in guiding such processes [136]. Especially, the thermodynamic metastability of the tumor cells is largely governed by its interaction with the microenvironment in the form of adherence to a substrate or by mechanical confinement of the surrounding extracellular matrix. The equilibrium between metabolic stability and cell adaptability determines phase transition processes and metastability. Sensitivity to the presence of growth factors or cytokines and alterations in cytoskeletal contractility of the cells within the tissues all contribute to cancer cell reactivation and tumor outgrowth [137,138]. Thus, instigating tumor cell dormancy allows clonal evolution and cell adaptation, resulting in a stronger and more developed tumor. This point of view can be supported by a recent experiment demonstrating the relationship between clonal diversity and tumor development [139]. Using genetically engineered tumor cells with cellular barcoding, scientists successfully tracked clonal diversity among the cancerous cell population during tumor regression, dormancy, and relapse. The study points out that only a certain population survives after oncogene withdrawal, and that a decrease in clonal diversity was observed in residual tumors, suggesting the possibility of a process of selection even while in the dormant state. Significantly, cell–matrix contact-mediated ERK activation via β1-integrin signaling plays potent roles to enable dormant cells re-entering the cell cycle [140,141]. These important findings suggest that the physical framework of the microenvironment should be considered as one important mechanism for tumor regression, cancer dormancy, cell clone evolution, and metastatic reactivation.

## 8. Seesaw Effects between PFKFB3 and Autophagy

**Figure 5:**
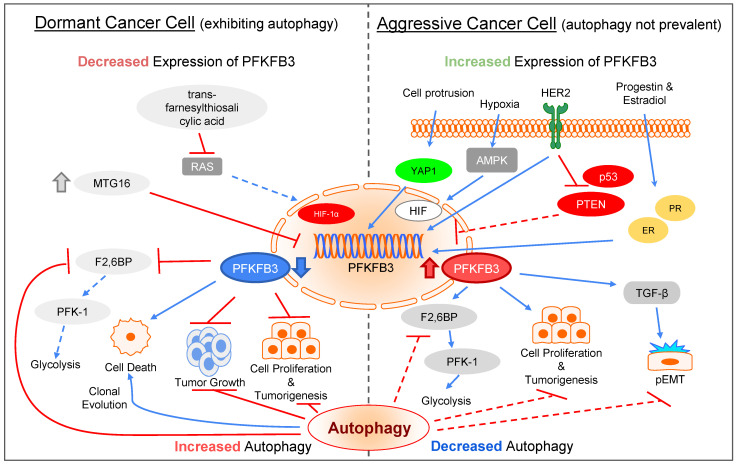
**Key roles of PFKFB3 expression in ECM stiffness-induced autophagy through changes in glucose metabolism.** (**Left** panel) In dormant cancer cells, autophagy is activated through several ways: (i) trans-farnesylthiosalicylic acid inhibits the RAS pathway, leading to the downregulation of HIF-1α and subsequent PFKFB3 expression; (ii) upregulation of MTG16, a transcriptional repressor functioning as a tumor suppressor, reducing PFKFB3 expression. PFKFB3 downregulation inhibits tumor growth, cell proliferation, and tumorigenesis, and promotes cell death. In addition, the activity of F2,6BP can be also inhibited, leading to downregulation of PFK-1 and glycolysis. (**Right** panel) During cancer development or dormancy reactivation, autophagy is not prevalent through several means that can upregulate PFKFB3 expression: (i) mechanosensing through cell protrusion; (ii) hypoxia-induced HIF activation through AMPK pathway; (iii) Her2 overexpression; (iv) inactivation of p53 and PTEN; (v) progestin- and estradiol-triggered nuclear receptor activation. PFKFB3 upregulation can further promote pEMT and cancer metastasis via TGF-β signaling. Cell proliferation and tumorigenesis can be also enhanced. Glycolysis activity can be recovered by activating F2,6BP and PFK-1.

Dormant cancer cells have been proven to exhibit autophagic behaviors while in a nonproliferative, unaggressive state. Autophagy and 6-phosphofructo-2-kinase/fructose-2,6-bisphosphatase 3 (PFKFB3) are both essential to the process of tumor progression, dormancy, and re-emergence (Figure 5). Studies have found that either HER2 expression or the loss of p53 and PTEN increases PFKFB3 expression. When PFKFB3 is activated, it presents strong kinase activity and promotes the synthesis of fructose-2,6-bisphosphate (F2,6BP). This can subsequently trigger phosphofructokinase-1 (PFK-1) and upregulate glycolysis flux [142], a phenomenon termed the “Warburg effect.” Hypoxia, progestin, and estradiol are also stimuli of PFKFB3, which promotes tumorigenesis and cell proliferation. Interestingly, mechanosensing through cell protrusion by F-actin has been recently reported to be able to upregulate PFKFB3 via YAP1 binding to a TEAD1/4 motif [143]. PFKFB3 is also a key effector protein to TGF-β, which has been proven to be an EMT inducer in tumor cells, aiding the metastasis stage of tumor development. Conversely, suppression of PFKFB3 can be achieved by inhibiting the Ras pathway, which downregulates HIF-1α [144], leading to glycolysis shutdown and cell death [145]. This pattern displayed by PFKFB3 is in an inverse relationship to the effect of autophagy on cancerous cells [146]. Further studies demonstrate that suppression of glycolysis due to the lack of PFKFB3 results in enhanced autophagy activation. Moreover, PFKFB3 inhibition retards the growth of a tumor, an observation that is in line with the expression of autophagy amongst cancer cells. This intricate “seesaw” effect between autophagy expression and PFKFB3 inhibition, and vice versa. Notably, cell adhesion and cytoskeletal organization have been recently found to participate in the control of the seesaw effects [147]. Through F-actin bundling and stress fiber formation during metastatic reactivation, the cytoskeleton networks in cancer cells gain the resistance to respond to mechanical cues from TME. This process can sequester TRIM21, the E3 ligase that can degrade PFKP, in the stress fiber, allowing persistence of high glycolytic rates in cancer cells [148].

## 9. Roles of pEMT and the Tumor Microenvironment in Tumor Progression

**Figure 6:**
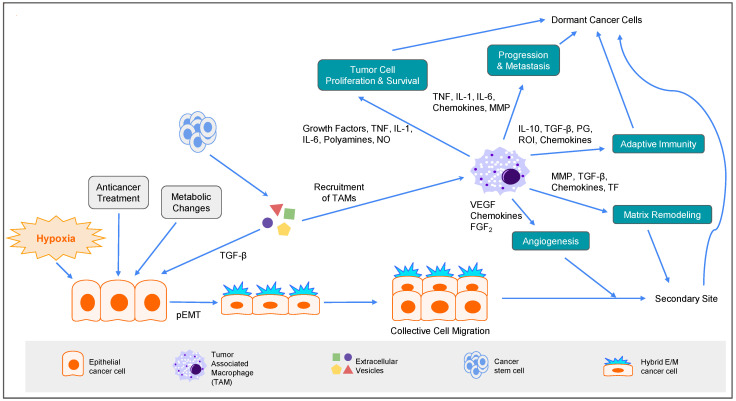
**Cellular plasticity of tumor cells is governed by partial EMT (pEMT).** Several factors are involved in the regulation of MET-pEMT-EMT transition and TME remodeling during tumor progression, including hypoxia, anticancer treatment, metabolic changes, and TGF-β secreted by cancer stem cells. These factors guide tumor cells in going into cellular transformation from an epithelial program to a partial epithelial-to-mesenchymal transition (pEMT) program. Cancer cells with the pEMT phenotype provide the necessary cell–cell contact for collective cell migration toward a secondary site. On the other hand, extracellular vesicles secreted by cancer stem cells recruit tumor-associated macrophages (TAMs), which can further assist cellular plasticity during metastasis by secreting a variety of cytokines and chemokines. Examples include angiogenesis and cluster cell migration promoted by VEGF and FGF2; TME remodeling for a protumor microenvironment by MMPs, TGF-β, and certain chemokines; cell proliferation and survival by TNF, IL-1, IL-6, polyamines, and NO; cancer progression and metastasis by TNF, IL-1, IL-6, MMPs; adaptive immunity by IL-10, TGF-β, PG, ROI, and certain chemokines; and cancer dormancy.

Cancer cells perform partial EMT (pEMT) during metastasis. This process is induced by a myriad of factors, including hypoxia, cytokines, anticancer treatment, and metabolic changes. More interestingly, pEMT is responsible for the disruption in cell-to-cell adhesion, cellular polarity, changes in cell–matrix adhesion, and remodeling of the cytoskeleton. The importance of the pEMT reaction is its aid in disseminating cancerous cells at an early stage. A notable example of this is in Her2+ breast cancer cells, where Her2+ early-disseminated cancer cells activated a Wnt-dependent EMT. [149] The process allows for cancerous cells to travel in clusters, and the benefit of a partial transition rather than a full transition offers the cancerous cells an option to revert to their epithelial state through the mesenchymal-to-epithelial transition (MET). The effects and causes of pEMT are in line with the proposed ideas described above. Anticancer treatment for the originally-diagnosed tumor activates pEMT, which aids in the stiffening of the extracellular matrix through changes in cell–matrix adhesion. This then allows for glucose metabolic changes through the YAP/TAZ pathway, which reactivates the pEMT pathway, resulting in multiple dissemination attempts from cancer cells, increasing the chances of a successful metastasis and/or metastasis to multiple secondary sites (Figure 6).

The tumor microenvironment aids prosperity of the tumor. Tumors do this by converting immune system cells into protumor cells. This is achieved through the secretion of chemokine ligands (CCL), such as CCL2, CCL3, CCL4, CCL5, and CCL8, as well as VEGF, colony-stimulating factor 1 (CSF-1), macrophage migration inhibitory factor (MIF), IL10, IL4, and TGF-β. Each of these extracellular vesicles serves a different purpose. A considerable portion of these extracellular vesicles are directed towards the cancer-associated fibroblasts (CAFs). The CCLs, CSF-1, and VEGF allow cancerous cells to gain control over macrophages and monocytes in the tumor microenvironment. This is the “recruitment” stage. CSF-1 and VEGF ensure the longevity of macrophages and monocytes, the “survival” stage, and TGF-β, along with IL4 and IL10, is used for macrophage differentiation. During these processes, VEGF induces angiogenesis, CSF-1 mediates the biological effects of macrophages, and MIF represses immune cells. Then, tumor cells gain the power to proliferate, perform matrix remodeling, encourage adaptive immunity, support angiogenesis, and promote tumor progression and metastasis. [150] These alterations in the immune system not only provide tumor cells the edge to prosper, but also provide the opportunity for cancer dormancy. Through the secretion of TGF-β and the conversion of tumor-associated macrophages and CAFs, tumors gain the ability to stiffen the extracellular matrix. This process creates a hypoxic environment, engaging another vicious cycle of pEMT, while inducing autophagy that furthers the efforts of tumor recurrence (Figure 6).

## 10. Conclusions

Enormous research progress into the biological impacts of the cytoskeleton network on mechanotransduction has advanced our understanding of molecular mechanisms in different steps of cancer progression. Alterations in the cytoskeleton network may serve as a newly defined driving force in carcinogenesis, contributing to changes in both cancer cell behaviors and biophysical/biochemical features of the TME. DNA fragmentations/damage induced by uneven mechanical forces can further generate a TME with long-term inflammation, leading to an induction of autophagy and cancer dormancy. Of note, cancer cells in the quiescent state can undergo clonal selection/evolution through cell metabolism reprogramming. In the near future, more effort should be made to further elucidate the regulatory mechanisms of communications among cancer cells, ECM and immune cells/stromal cells in the TME, and to define the corresponding novel targets for mechanotransduction signaling, especially the ones in involved in the regulation of cytoskeletal dynamics, the mechanical interplay between cellular processes, and the role of environmental cues. Finally, how to apply these findings to develop new strategies that can determine cancer cell fate opens an important avenue to explore future therapeutic options.

## Data Availability

Not applicable.
